# Comprehensive immune landscape of lung-resident memory CD8^+^ T cells after influenza infection and reinfection in a mouse model

**DOI:** 10.3389/fmicb.2023.1184884

**Published:** 2023-06-21

**Authors:** Ju Jia, Hui Li, Zhisheng Huang, Jiapei Yu, Ying Zheng, Bin Cao

**Affiliations:** ^1^Graduate School of Peking Union Medical College, Chinese Academy of Medical Sciences, Peking Union Medical College, Beijing, China; ^2^Department of Pulmonary and Critical Care Medicine, Center for Respiratory Diseases, China-Japan Friendship Hospital, Beijing, China; ^3^Department of Respiratory Medicine, The First Affiliated Hospital of Nanchang University, Nanchang, China; ^4^National Clinical Research Center for Respiratory Diseases, Clinical Center for Pulmonary Infections, China-Japan Friendship Hospital, Capital Medical University, Beijing, China

**Keywords:** influenza virus, lung, T cell memory, CD8^+^ tissue resident memory T, reinfection

## Abstract

**Background:**

Resident phenotypic memory CD8^+^ T cells are crucial for immune defense against pathogens. However, little is known about the potential transitions and regulation mechanisms of their function after influenza virus infection and reinfection. In this study, we utilized integrated transcriptome data and *in vivo* experiments to investigate the key characteristics behind it.

**Methods:**

Two single-cell RNA sequencing (scRNA-seq) datasets of lung CD8^+^ T cells and one RNA-seq dataset of lung tissue after infection or reinfection were included. After Seurat procedures classifying CD8^+^ T subsets, the scCODE algorithm was used to identify the differentially expressed genes for GSVA, GO, and KEGG pathway enrichment. Monocle 3 and CellChat were used to infer pseudotime cell trajectory and cell interactions. The ssGSEA method was used to estimate the relative proportions of immune cells. The findings were confirmed with a mouse model via flow cytometry and RT-PCR analysis.

**Results:**

Our study refined the landscape of CD8^+^ T-cell subsets in the lung, showing that CD8^+^ Trm cells accumulated in the lung within 14 days after influenza infection. The classical CD8^+^ Trm cells co-expressed a high level of CD49a and even maintained 90 days after primary infection. The ratio of CD8^+^ Trm cells decreased 1 day after influenza reinfection, which may be parallel with their potential transition into effector types, as observed in trajectory inference analysis. KEGG analysis suggested that PD-L1 expression and PD-1 checkpoint pathway were upregulated in CD8^+^ Trm cells on day 14 after infection. GO and GSVA analyses revealed that PI3K-Akt-mTOR and type I interferon signaling pathways were enriched in CD8^+^ Tem and Trm cells after reinfection. Additionally, CCL signaling pathways were involved in cell interaction between CD8^+^ Trm cells and other cells, with Ccl4-Ccr5 and Ccl5-Ccr5 ligand/receptor pairs being important between CD8^+^ Trm and other memory subsets after infection and reinfection.

**Conclusion:**

Our data suggest that resident memory CD8^+^ T cells with CD49a co-expression account for a large proportion after influenza infection, and they can be rapidly reactivated against reinfection. Function differences exist in CD8^+^ Trm and Tem cells after influenza infection and reinfection. Ccl5-Ccr5 ligand/receptor pair is important in cell interactions between CD8^+^ Trm and other subsets.

## 1. Introduction

Influenza is still a major public health burden and continues to cause up to 5 million cases of severe illness and 650,000 deaths annually around the world (Paules et al., 2018; van de Wall et al., 2021). Persistent mutations of the influenza virus have posed a great challenge to vaccines that rely on the induction of strain-specific antibodies, which could provide little to no protection against mismatched viruses. T cells recognize internal and more conserved parts of the influenza virus that are far less prone to mutation (Yewdell et al., 1985; Belz et al., 2000). Therefore, more and more researchers are paying attention to T-cell immunity (Pizzolla and Wakim, 2019).

Protection of T-cell response mainly depends on immunological memory, which can respond more rapidly and effectively to pathogens if they are encountered again (Zheng and Wakim, 2022). Memory T cells can be broadly categorized into three main populations: central memory T cells (Tcm), effector memory T cells (Tem), and tissue-resident memory T cells (Trm) based on phenotype, localization, and function (Yuan et al., 2021). In contrast to the circulating subsets, Trm cells stably locate along the respiratory tract characterized by T-cell activation marker CD69 and integrins such as CD103 and/or CD49a (Topham and Reilly, 2018). CD8^+^ Trm cells are considered the first line of defense in peripheral tissues against pathogens (Yuan et al., 2021). CD8^+^ Trm cells can produce chemokines after local tissue activation and recruit non-antigen-specific T cells to exert natural effector functions (Schenkel et al., 2013; Ariotti et al., 2014). Recently, it has been illustrated that mice with CD103^+^ Trm cells in the lung exhibit more rapid viral titer decline and lighter weight loss following heterosubtypic infection (Wu et al., 2014; Schmidt and Varga, 2018).

Due to the crucial role of CD8^+^ T cells residing in the lung, especially Trm cells, in protecting against pathogen re-encounter, it is vital to investigate the dynamic changes and functional transformation of these cells during the memory phase after infection and the early stages of reinfection. Identifying key factors and potential roles of CD8^+^ Trm cells in these processes is of the utmost importance. It has been indicated that lung Trm cells show high baseline levels of mRNA encoding inflammatory signals, such as granzyme B, IFNγ, and TNF, during homeostasis, even without stimulation (Hombrink et al., 2016; Oja et al., 2018; Lange et al., 2022). Furthermore, CD8^+^ Trm cells can rapidly release abundant inflammatory cytokines and chemokines including IFNγ, TNFα, CCL-3, and CCL-4 and cytotoxic granules such as perforin and granzyme B upon reactivation (Schenkel et al., 2013; Ariotti et al., 2014; Hasan and Beura, 2022). However, the characteristics of CD8^+^ Trm cells described above are mainly related to effector phenotype cells, whether the cell state can be transformed in immune responses against reinfection has not been explicated. Additionally, the roles of the chemokines mentioned above in resident memory cells still need to be uncovered.

In this study, single-cell RNA sequencing (scRNA-seq) and bulk RNA sequencing (bulk RNA-seq) analysis were employed to present a comprehensive landscape and delicate details of the transcriptomic profiles. We applied scRNA-seq data of mice lungs to characterize the CD8^+^ cell subsets, especially CD8^+^ Trm cells, with gene expression characteristics, developmental trajectories, and cell communication post-influenza infection and reinfection. Meanwhile, we used publicly available bulk RNA-seq data to analyze immune cell infiltrations between first infection and reinfection. We further confirmed the findings with a mouse model via flow cytometry, enzyme-linked immunosorbent assay (ELISA), and RT-PCR analysis.

## 2. Materials and methods

### 2.1. Data collection and preprocessing

We obtained the datasets GSE183890, GSE186839, and GSE194058 from the Gene Expression Omnibus (GEO) database (http://www.ncbi.nlm.nih.gov/gds/). We converted the RNA-seq dataset to transcripts per kilobase million (TPM) for further analysis. The scRNA-seq data in GSE186839 consists of CD8^+^ T cells isolated via fluorescence-activated cell sorting (FACS) from mouse lung samples of day 0 (D0), day 7 (D7), and day 14 (D14) after influenza infection.

We also downloaded the annotation expression matrix of the GSE194058 dataset which was processed in published article (MacLean et al., 2022). This dataset included the expression profiling of CD45^+^ cells in the lungs at 42 days post-primary infection and 1 day after reinfection (referred to as resting and rechallenged in the analysis; shown in Supplementary Figures 1A, B). We also analyzed dataset GSE183890 for immune cell infiltrations, and processing details are shown in Supplementary methods.

All of these datasets were imported and processed using the R package Seurat. Details of the included datasets can be found in Supplementary Tables 1, 2.

### 2.2. scRNA-seq data processing

First, we applied quality control standards to remove low-quality cells from the Seurat object that included data in GSE186839. Processing details are shown in Supplementary methods. In addition, we created a Seurat object for CD8^+^ T cells in GSE194058 by extracting data from an h5ad file that contained annotated information (Supplementary Figures 1A, B).

All integrated data were normalized, scaled, and processed using principal component analysis (PCA). We further visualized the data and clustered it using the T-distribution stochastic neighbor embedding (t-SNE) and uniform manifold approximation and projection (UMAP) methods.

We identified subpopulations of CD8^+^ T cells by examining the expression of canonical markers, including *Cd3d, Cd3e, Cd4*, and *Cd8a*, to separate CD8^+^ T cells from other cell types. We next used *Ccr7, Sell* (CD62L), *Il7r* (CD127), *Klrg1*, and *Cd44* to separate naive and effector T cells (Eff) from memory T cells. Furthermore, we combined *Cxcr3, Itgae* (CD103), *Cd69*, and *Itga1* (CD49a) to divide memory T cells into classical Tcm, Tem, and Trm subsets; and *Gzma, Gzmb, Lag3, Pdcd1, Mki67*, and *Stmn1* to portray details.

### 2.3. Detection of differentially expressed genes and pathway analysis

Differentially expressed gene (DEG) analysis was conducted using the R package scCODE (Zou et al., 2022) and selected with the thresholds of fold change (FC) > 0.7 and an adj. *p*-value of < 0.05. Furthermore, gene ontology (GO) categories and the Kyoto Encyclopedia of Genes and Genomes (KEGG) pathways were analyzed using the enrichGO and enrichKEGG functions in the R package ClusterProfiler (Yu et al., 2012), and a *p*-value of < 0.05 was considered statistically significant. Additionally, gene set variation analysis (GSVA) was performed to assess potential changes in pathway activity in each sample using the GSVA package based on gene collections hallmark (H) gene sets from the Molecular Signatures Database (MSigDB, https://www.gsea-msigdb.org/gsea/msigdb/index.jsp).

### 2.4. Analysis of infiltrating immune cells

The ssGSEA algorithm (Barbie et al., 2009) was performed to estimate the relative composition of different immune infiltrating cells based on mRNA expression data in immune gene sets, details shown in Supplementary methods. The gene sets used in this article were cited from Charoentong et al. (2017).

### 2.5. Trajectory analysis

To determine the trajectory of CD8^+^ T cells in scRNA-seq databases, we used Monocle 3 (Cao et al., 2019a). The data were processed, and a graph-based trajectory inference function was used to generate the trajectory tree. Cell clustering and annotation were performed using Seurat, as described earlier. Once the cells were ordered along the trajectory, genes were identified to describe function changes in pseudotime.

### 2.6. Cell–cell interaction analysis

To assess cell–cell interactions between CD8^+^ Trm cells and other cell populations, we utilized the recently developed CellChat (Jin et al., 2021) platform. Information flows for each signaling pathway, defined as the communication probabilities among all pairs of cell groups in the inferred network, were calculated and compared between conditions. In order to explore changes in specific signaling pathways underlying the global alterations, we analyzed and compared CD8^+^ Trm cell–cell communications with other subsets between different conditions.

### 2.7. Mice and reagents

Six to 8-week-old male wild-type C57BL/6J mice from Beijing Vital River Laboratory were used in all the experiments. All animal studies and procedures were approved by the Chinese Center for Disease Control and Prevention. Mice were anesthetized before intranasal (i.n.) inoculation with 1,500 plaque-forming units (PFU) of A/Puerto Rico/8/34 (PR8) for influenza primary challenge. For rechallenge, mice were reinfected with 10^7^ PFU of PR8. Virus titers were determined as previously described (Wu et al., 2014).

### 2.8. Preparation of single-cell suspensions and flow cytometry

To obtain the single-cell suspension, we cut the lungs into small pieces and incubated them with collagenase (125 U/ml) and deoxyribonuclease (0.1 mg/ml) for 1 h at 37°C. Next, we enriched the samples by centrifugation in a 40/70% Percoll gradient to isolate lymphocytes. To assess Trm cells, lung samples were analyzed by flow cytometry. An intravascular (iv.) stain with anti-CD8b antibody was applied prior to euthanasia to distinguish CD8^+^ T cells in the tissue (iv. ^−^) from those remaining in small capillary beds (iv. ^+^).

After blocking unspecific binding with αCD16/32, the fluorophore-conjugated antibodies in this study included CD3, CD8a, CD44, CD62L, CD69, CD103, CD49a, PD-1, CCL5, and CCR5 from BioLegend or Tonbo. The samples were analyzed using a CytoFLEX LX (Beckman Coulter), and data analysis was conducted using FlowJo v10 software (TreeStar).

### 2.9. RNA extraction and RT-PCR analysis

RNA was extracted from the homogenized lung tissue, and 1 mg of purified RNA was then reversely transcribed into cDNA. The cDNA was then subjected to RT-PCR analysis using SYBR Green, with other processing procedures listed in the Supplementary methods. The results were normalized to the expression of glyceraldehyde-3-phosphate dehydrogenase (GAPDH). The relative mRNA expression level was quantified using the 2^−Δ*ΔCt*^ method. The relative levels of mice Ccr5, Ccl5, and Ccl4 were detected with reverse transcription primers, listed in Supplementary Table 3.

### 2.10. Chemokines detection

To determine the levels of Ccl4, Ccl5, and Ccr5 in the lung supernatants collected from the experiments described above, ELISA kits from Multi Sciences and ELK Biotechnology were used. The detection limits were 16 pg/ml for Ccl4 and Ccl5 and 0.16 ng/ml for Ccr5.

### 2.11. Statistical analysis

Unpaired two-tailed Student's *t*-tests were used to evaluate simple two-group comparisons. ^*^P < 0.05, ^**^P < 0.01, ^***^P < 0.001, and ^****^P < 0.0001 represented significant statistical differences. Statistical analysis of data was performed using GraphPad Prism 8 (GraphPad Software Inc.).

## 3. Results

### 3.1. The immune landscape of CD8^+^ T cells during the memory phase after influenza infection

**Figure 1:**
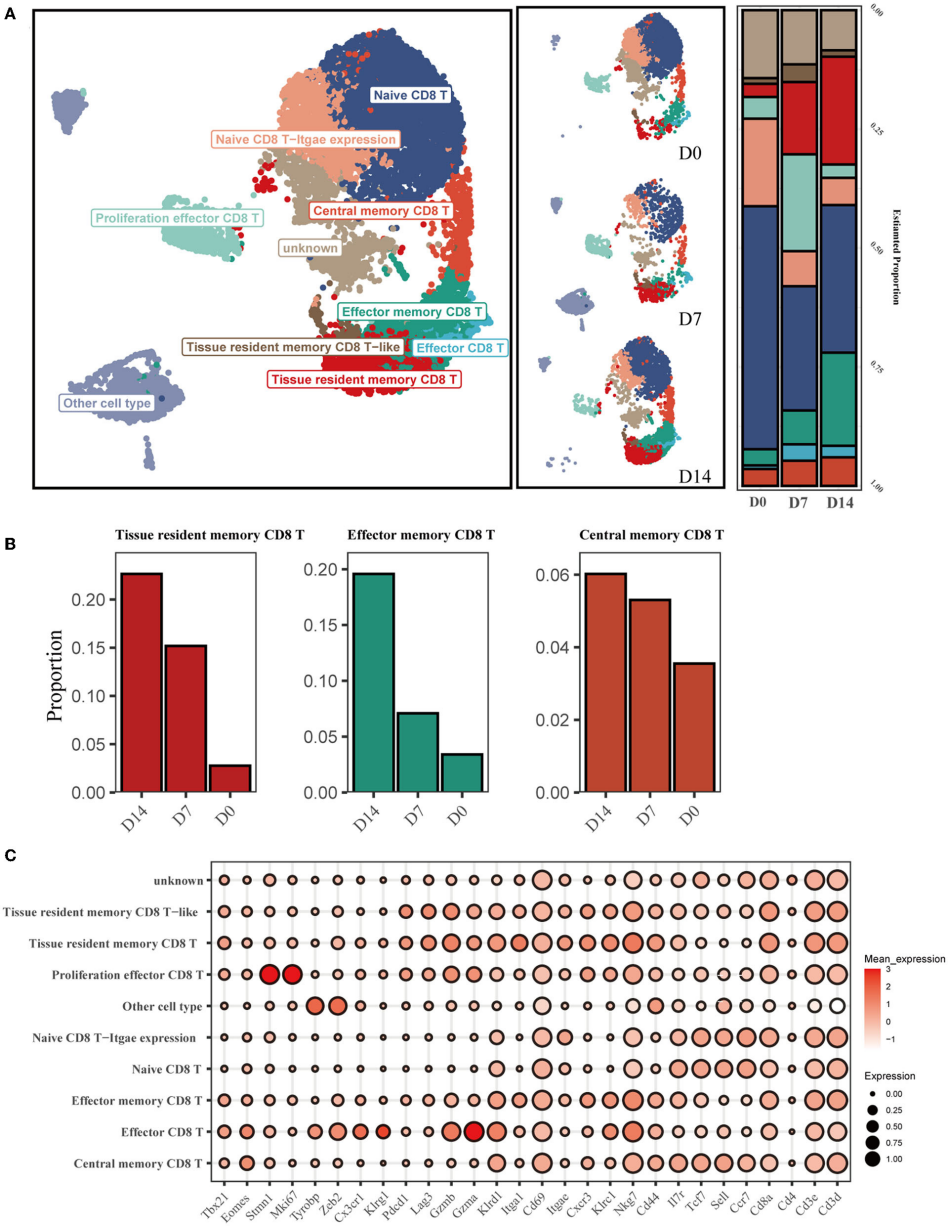
Single-cell transcriptomic analysis of CD8^+^ T cells after influenza infection. **(A)** UMAP map presents major CD8^+^ T-cell subsets in the left panel and the composition of major T-cell subsets of three conditions in the middle panel. The bar plot displays the proportion of cell types separated by conditions in the right panel. **(B)** Three main memory cell types (tissue-resident memory, effector memory, and central memory) are presented in separate graphs along with their respective proportion under different conditions. **(C)** The dot plot shows the expression distribution of canonical cell markers in CD8^+^ T-cell subsets after annotation in **(A)**.

To investigate the dynamic characteristics of memory CD8^+^ cells in the recovery phase, with a particular focus on the tissue-resident memory CD8^+^ T subsets (CD8^+^ Trm), we analyzed scRNA-seq data from GSE186839, which included CD8^+^ T cells from day 0 (D0), day 7 (D7), and day 14 (D14) after influenza infection. Eight transcriptionally distinct clusters using known cell-type markers were identified and two subpopulations could not be identified as any canonical cell type according to a combination of different marker genes (Figures 1A, C). The proliferation effector CD8 T cells were characterized with *Mki67* and *Stmn1* expression. Naive CD8 T-Itgae expression cells were consistently marked by *Sell, Ccr7*, and *Cd44*, and enriched *Itgae*.

We then compared the proportion of each cell type across D0, D7, and D14 (Figure 1A). The proportion of infiltrating naive T cells (NT) decreased markedly at D7 and D14 compared to D0, while the proportion of effector cells (Eff) increased from 0.75% at D0 to 3.4% at D7. CD8^+^ Trm and Tem cells exhibited a substantial increase at D14 (Figure 1B).

### 3.2. The immune landscape of CD8^+^ T cells during the early phase post-influenza reinfection

**Figure 2:**
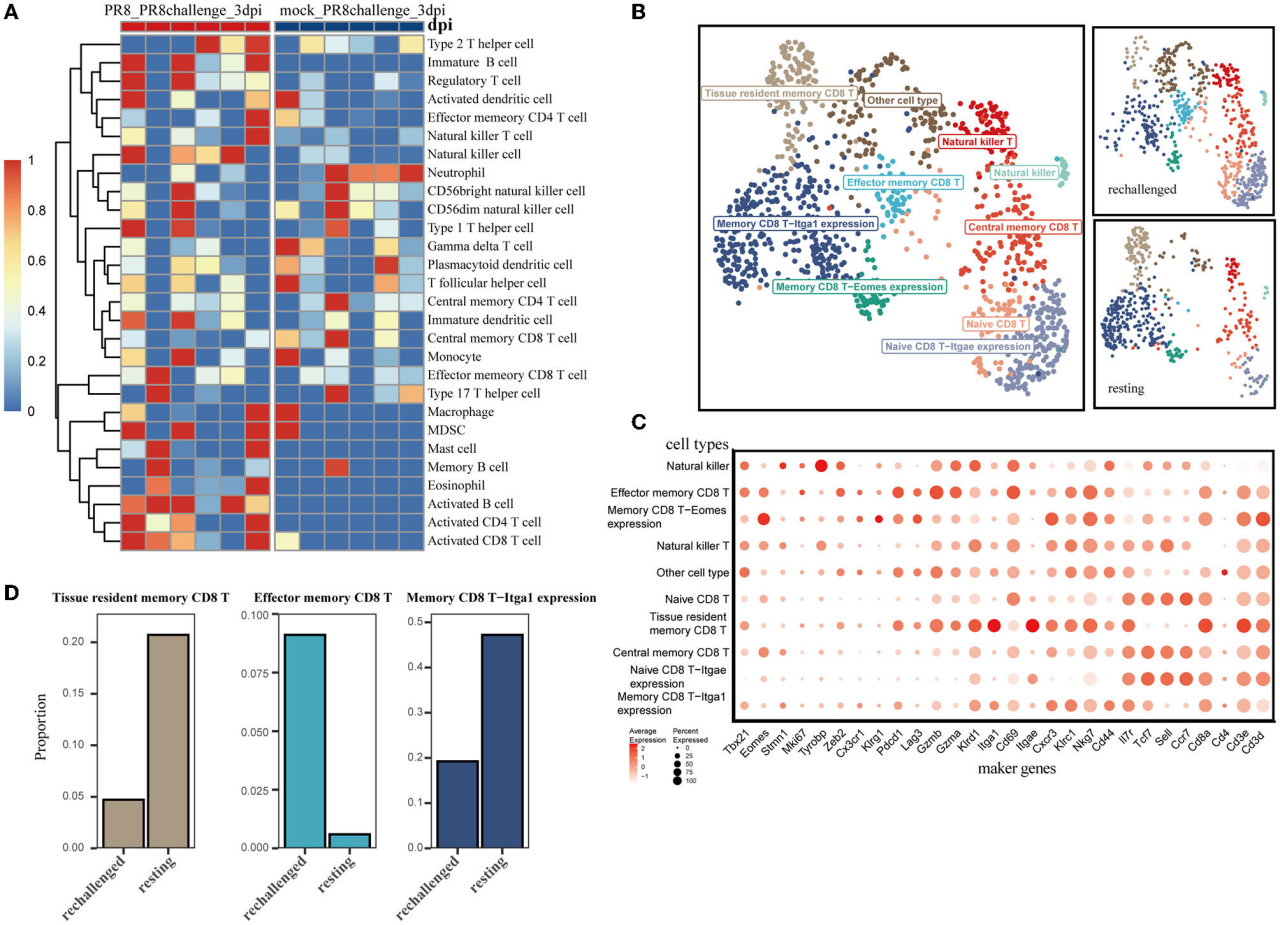
Single-cell transcriptomic analysis of CD8^+^ T cells after reinfection. **(A)** ssGSEA was performed to estimate the relative proportions of cell subpopulations in PR8_PR8challenge_3dpi and mock_PR8challenge_3dpi samples. **(B)** CD8^+^ T-cell clusters are colored by cell-type annotation in UMAP: the total corresponding cells (left panel) and the different conditions (rechallenged and resting in the right panel). **(C)** The dot plot shows the expression distribution of canonical cell markers in CD8^+^ T-cell subsets in **(B)**. **(D)** Three main memory cell types (tissue-resident memory, effector memory, and CD49a expression memory) are presented in separate graphs along with their respective proportion under different conditions.

To explore the difference in infiltrating immune cells, especially CD8^+^ T cells, during the early phase of first infection and post-influenza reinfection, we used the ssGSEA algorithm on the transcriptome data of lung samples collected 3 days post infection and reinfection (GSE183890). We found that activated CD8 T cells were differentially distributed in reinfection compared with primary infection (Figure 2A and Supplementary Figure 1E), which suggests that CD8^+^ T cells play a critical role during the early phase of reinfection.

Using the annotated GSE194058 dataset, which included lung CD8^+^ T cells at 42 days after the first infection (resting) and 1 day after reinfection (rechallenge), we proceeded to characterize CD8^+^ T cells during reinfection, which were further classified into nine cell types (Figures 2B, C). In addition to the validated cell types, memory CD8 T-Itga1 expression cells were characterized by high *Itga1* and low *Itgae* expression profiles. Natural killer CD8 T cells were marked by relatively high expression of *Tyrobp* and sparse expression of *Cd8a* (Figure 2C). With this dataset, we discovered that memory CD8 T-Itga1 cells and CD8^+^ Trm were important parts after infection, while the proportion of them decreased 1 day after the influenza rechallenge (Figure 2D). Both of them presented with high-level expression of CD49a (*Itga1*). Recent studies have indicated that CD8^+^ Trm cells expressing CD49a, CD103, and CD69 are the predominant memory cell populations in the lung (Kumar et al., 2017). The co-expression of CD49a on CD8^+^ Trm cells is a crucial characteristic of the resident phenotype T cells.

### 3.3. Functional differences and transformations of CD8^+^ Trm cells after first influenza infection and reinfection

**Figure 3:**
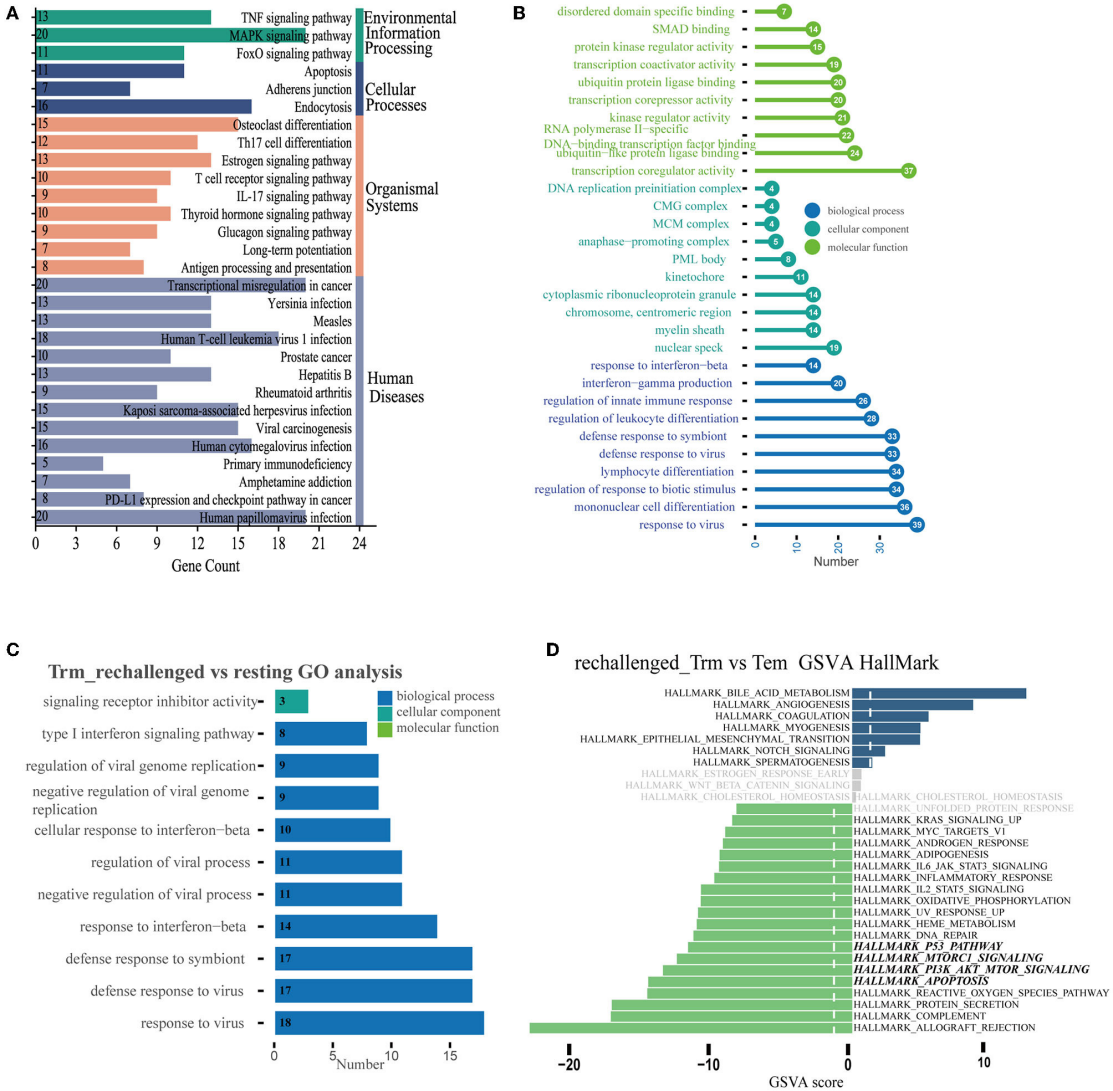
Differential expression pattern and functional enrichment analysis. **(A, B)** Plots represent the enriched pathways of KEGG and GO analyses of significant DEGs in Trm between D14 and D7. **(C)** GO terms enriched in CD8^+^ Trm cell between resting and rechallenged. **(D)** Differences in pathway activities scored per cell by GSVA in the hallmark databases between CD8^+^ Trm and Tem cells in rechallenged.

We further explored the dynamic functional changes in CD8^+^ Trm cells after influenza infection and identified 600 differently expressed genes in CD8^+^ Trm cells between D14 and D7 based on the defined criteria. Top 10 upregulated genes in CD8^+^ Trm cells at D14 were *Itgae, Cdh1, Csf1, Krt83, Rgcc, Ly6g5b, 2900026a02rik, Itsn1, Klf4*, and *Gm36723* (Supplementary Figure 1D). The upregulated DEGs were mainly enriched in the FoxO signaling pathway, apoptosis, PD-L1 expression, PD-1 checkpoint pathway, and adherens junction in KEGG (Figure 3A). Additionally, we performed GO enrichment analysis and found that response to the virus, interferon-gamma production, and regulation of innate immune were enriched and gradually upregulated in CD8^+^ Trm cells during the memory phase (Figure 3B).

While during the early stage of reinfection, the most significant enriched GO terms of 31 upregulated DEGs in CD8^+^ Trm cells were related to the response to the virus, type I interferon signaling pathway, and interferon-beta (Figure 3C), which suggests that CD8^+^ Trm cells play a critical role in fighting against influenza reinfection. We then investigated the functional differences between CD8^+^ Trm and Tem cells after reinfection. Our analysis showed that CD8^+^ Tem cells exhibited heightened activities related to protein secretion, apoptosis, PI3K-Akt-mTOR signaling, Mtorc1 signaling, p53 pathway, and DNA repair (Figure 3D).

### 3.4. Pseudotime trajectory inference analysis of CD8^+^ T-cell populations after first infection and reinfection

**Figure 4:**
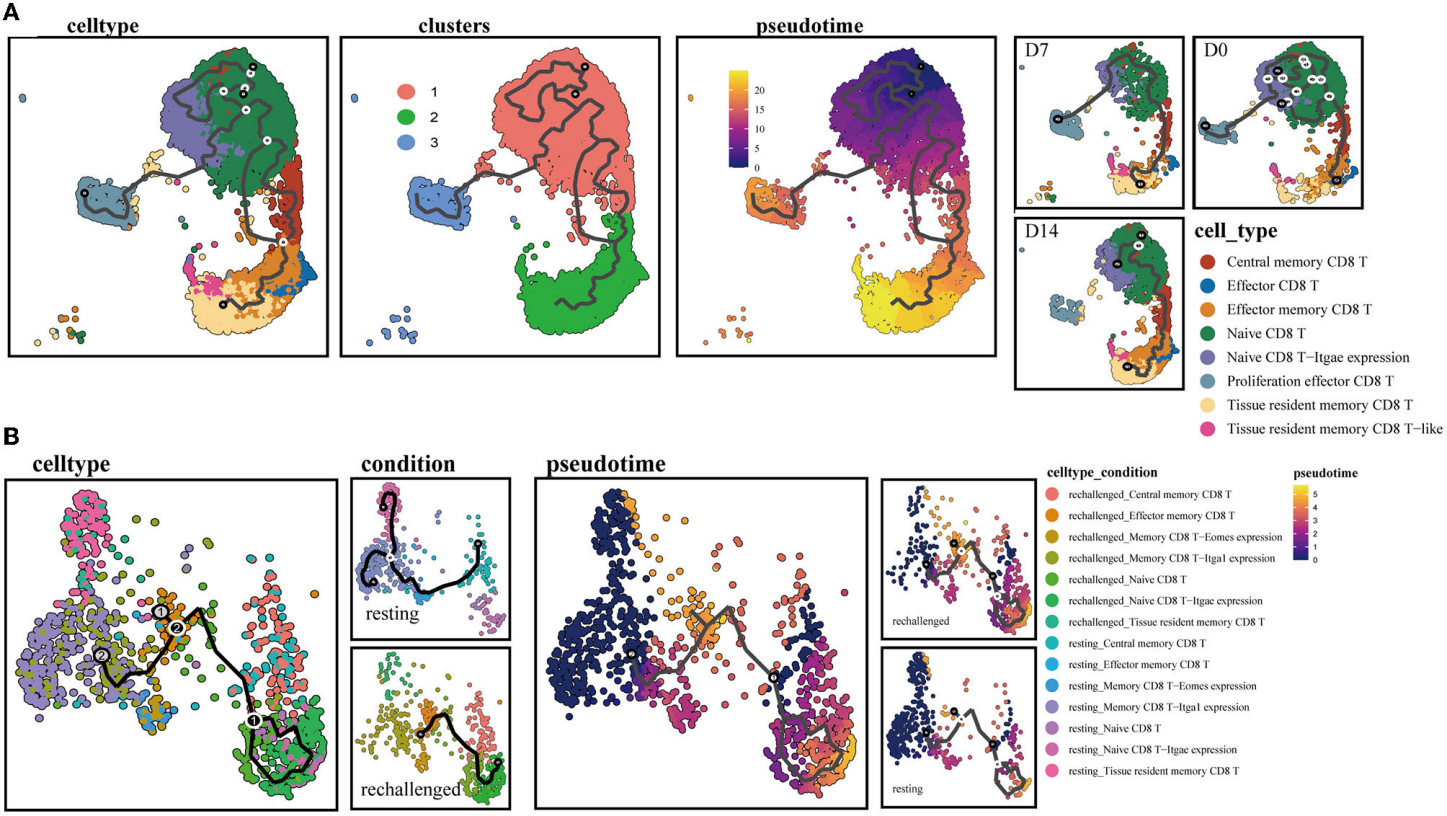
Single-cell trajectory analysis of memory CD8^+^ T clusters. **(A)** UMAPs show pseudotime reconstruction and developmental trajectory of CD8^+^ T cells of D0, D7, and D14 by Monocle 3. Cells are colored based on the cell types, clusters, predicted pseudotime, and conditions. **(B)** Pseudotime reconstruction and developmental trajectory of total CD8^+^ T cells under resting and rechallenge conditions were processed by Monocle 3. Cells are colored according to the cell types and predicted pseudotime.

To further explore the continuum of class-switching states in CD8^+^ T-cell subsets during the memory phase after infection and the early stages of reinfection, we conducted the pseudotime ordering of single cells. When analyzing the memory of CD8^+^ T cells during the first infection, we reconstructed a trajectory with three branches starting from NT cells (Figure 4A). Naive type CD8^+^ T cells had their respective developmental trajectories to other cell types. Notably, CD8^+^ Trm cells were almost exclusively located at the branch ends and were closely positioned behind CD8^+^ Tem cells (Supplementary Figure 2A). This might suggest a potential development from CD8^+^ Tem to Trm cells during the memory phase.

During reinfection, two trajectory branches were constructed. The left trajectory primarily included cells before reinfection such as CD8^+^ Trm and memory CD8 T-Itga1 expression cells, while the right trajectory mainly consisted of cells after reinfection, including CD8^+^ Tcm, NT. Both branches converged to CD8^+^ Tem cells, which were predominantly observed after reinfection (Figure 4B). The trajectory indicated that memory CD8^+^ T-cell subsets expressing canonical Trm markers or relatively high CD49a after infection have the developmental potential to become effector phenotype cells after reinfection.

### 3.5. Cell–cell interactions among subtypes of CD8^+^ T cells in the steady-state post-influenza infection and early stage after reinfection

**Figure 5:**
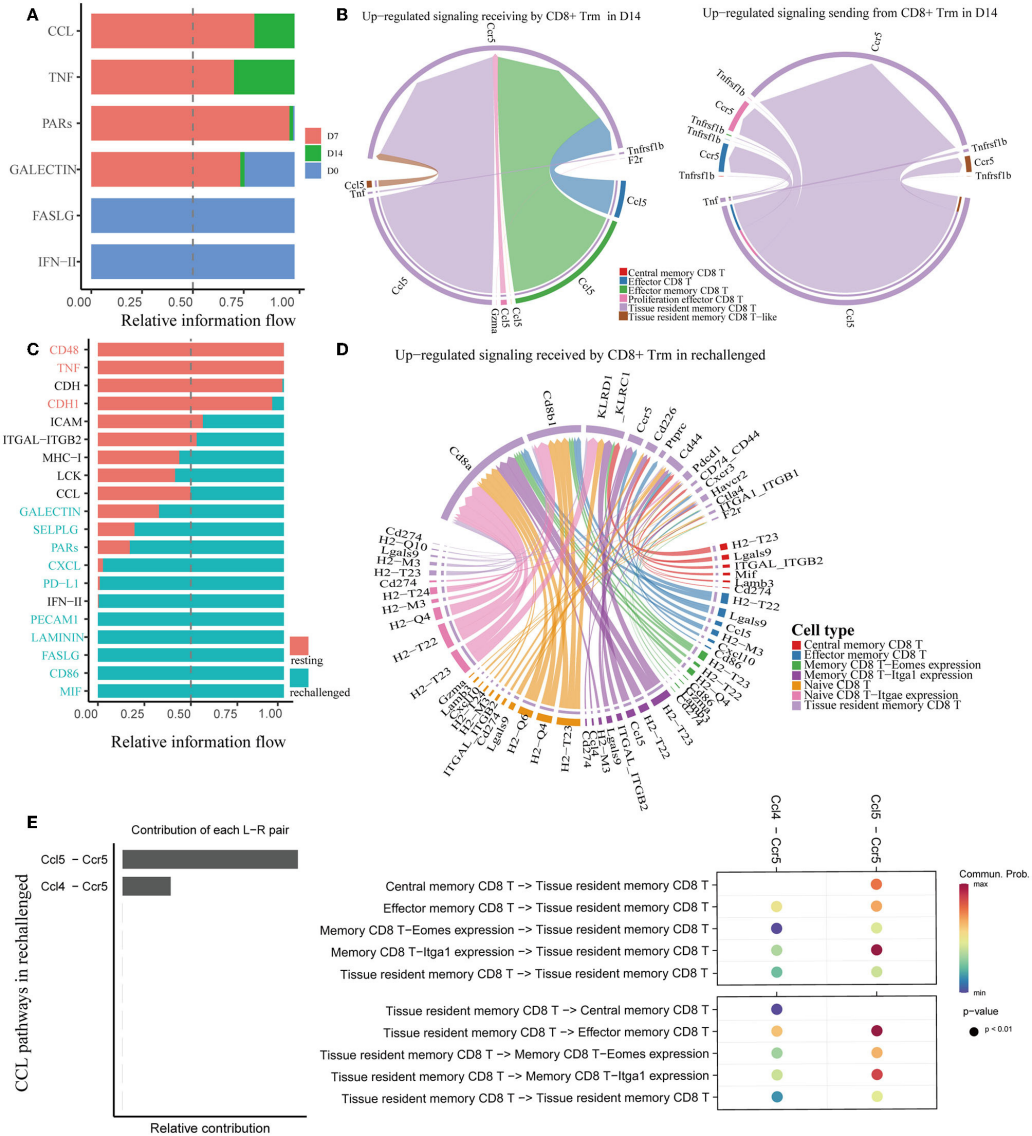
Analysis of cell–cell communication in memory CD8^+^ T cells. **(A, C)** All the significant signaling pathways are ranked based on their differences in overall information flow within the inferred networks between conditions. **(B)** Chord plots show the upregulated significant signaling pathways between Trm and other cell subsets in D14 compared to D7. **(D)** The plot shows the upregulated signaling pathways received by CD8^+^ Trm in rechallenged. **(E)** Plots show the relative contribution of CCL pathways **(left)** and communication probabilities mediated by CCL ligand–receptor pairs between CD8^+^ Trm and other subsets **(right)** in rechallenged.

We utilized CellChat to predict cell–cell communications associated with CD8^+^ Trm cells. Signaling pathways, such as TNF and CCL, were found to be involved at D7 and D14 after influenza infection (Figure 5A). Further investigation of specific ligand–receptor interactions between CD8^+^ Trm cells and other cell types revealed the importance of Ccr5-related signaling (Supplementary Figure 2B). Upregulated signaling in D14 compared to D7 was also identified, with CD8^+^ Trm cells receiving upregulated signals via pathways such as Ccl5-Ccr5 from CD8^+^ Tem, Eff subsets, and Trm cells themselves. Additionally, CD8^+^ Trm cells were found to regulate Eff via Ccl5-Ccr5 (Figure 5B).

Similar to the first infection, CCL signaling pathways were also revealed to be critically important in regulating CD8^+^ T cells during reinfection (Figure 5C). Ccl5-Ccr5 and Ccl4-Ccr5 were involved in the interaction between CD8^+^ Trm and other subsets during reinfection (Supplementary Figure 2C). Further analysis revealed that the Ccl5-Ccr5 signal was received by CD8^+^ Trm cells from Tem and memory CD8 T-Itga1 expression cells, and the Ccl4-Ccr5 signal was from memory CD8 T-Itga1 expression cells (Figure 5D).

We specifically extracted CCL pathways to visualize the correlations between CD8^+^ Trm and other subsets during reinfection (Figure 5E and Supplementary Figure 2D). Our analysis revealed that Ccl5-Ccr5 was relatively more important than Ccl4-Ccr5, and the communication probabilities were mainly within the memory CD8^+^ T-cell subsets. Notably, the communication probabilities of Ccl5-Ccr5 between CD8^+^ Trm and Tem, as well as between Trm and memory CD8 T-Itga1 expression cells, were more evident after reinfection.

### 3.6. Validation of observations related to influenza infection and reinfection in this study

**Figure 6:**
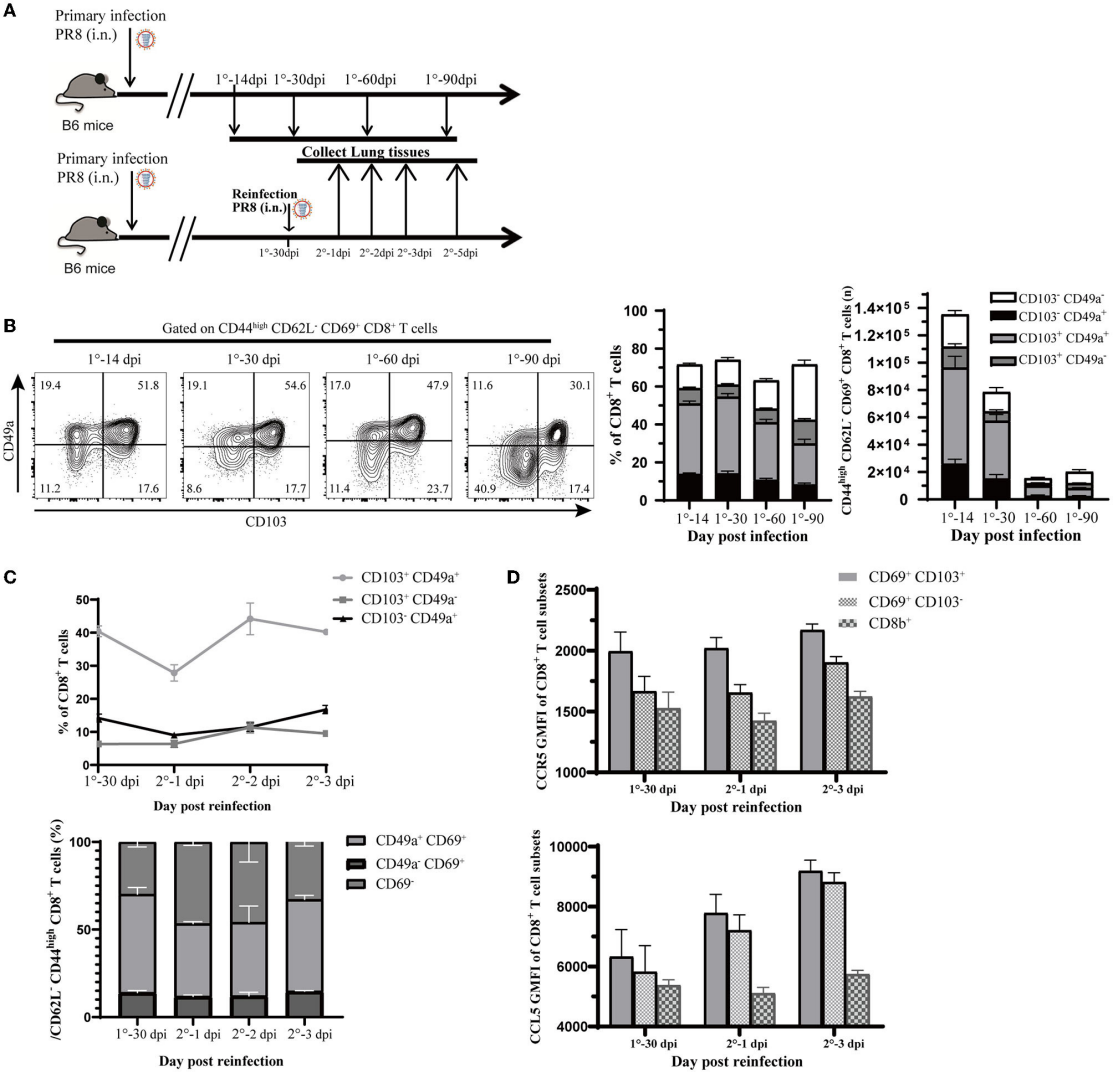
Validation of observations in this study relevant to influenza infection and reinfection. **(A)** Experimental design for validation. Mice were infected with 1,500 PFU PR8 virus i.n., and lung samples were analyzed at different time points (14, 30, 60, and 90 days post-infection). Mice were reinfected with 10^7^ PFU PR8, and data were collected at 0, 1, 2, 3, and 5 days post-reinfection. **(B)** Representative flow cytometry plots show the percentage of CD103 and CD49a expression in CD44^high^ CD62L^−^ CD69^+^ CD8^+^ T cells at the indicated time points. Stacked analysis shows the percentage and number of CD103^+^ CD49^+^, CD103^+^ CD49^−^, CD103^−^ CD49^+^, and CD103^−^ CD49^−^ CD44^high^ CD62L^−^ CD69^+^ CD8^+^ T-cell subsets. Data are presented as mean ± SEM (*n* = 5). **(C)** Line chart shows the percentage of CD103^+^ CD49^+^, CD103^+^ CD49^−^, and CD103^−^ CD49^+^ CD44^high^ CD62L^−^ CD69^+^ CD8^+^ T-cell subsets and stacked map displays percentage of CD49^+^ CD69^+^, CD49^−^ CD69^+^, and CD69^−^ CD8^+^ T-cell subsets in CD44^high^ CD62L^−^ CD8^+^ T cells. Data are presented as mean ± SEM (*n* = 5). **(D)** Ccr5 and Ccl5 expression assessed at 0, 1, and 3 days post-reinfection in Trm (CD69^+^ CD103^+^), Tem (CD69^+^ CD103^−^) from lung (iv. CD8b^−^), and CD8^+^ T cell in circulation (iv. CD8b^+^). Data are presented as mean ± SEM of five PR8 mice in each group. 1° represents primary infection; 2° represents reinfection; dpi refers to day post-infection; GMFI represents geometric mean fluorescence intensity; i.n. means intranasally. Statistical significances were analyzed with unpaired two-tailed Student's *t*-test. *P < 0.05, **P < 0.01, ***P < 0.001, and ****P < 0.0001.

To confirm the above findings in a mouse model of influenza virus infection, we infected C57BL/6J mice with PR8 intranasally (Figure 6A). Consistent with findings based on scRNA-seq analysis, via flow cytometry (Supplementary Figure 3A), we found that CD8^+^ Trm cells that expressed CD49a were a significant portion of both memory and total CD8^+^ T cells in the lung tissue (iv. CD8b^−^) after recovery from the infection. Memory CD8^+^ T cells that expressed high levels of CD49a, with sparse CD103 expression, were found to be an essential component of resident memory cells (Figure 6B). Moreover, the number and proportion of resident memory CD8^+^ T subsets were relatively higher at 14 days after infection (as shown in Figure 6B), but gradually declined during the memory phase. In addition, we observed that PD-1 expression increased in CD8^+^ Trm cells in the memory phase after infection (Supplementary Figure 3B).

Then, we rechallenged mice at 30-days post first infection to create a reinfection model with the PR8 virus (Figure 6A). We observed that the proportion of CD49a^+^ memory CD8^+^ T cells co-expressed with CD103 or CD69 had decreased at 1 day post-reinfection and rebounded quickly (Figure 6C). Meanwhile, the percentage of other subsets, such as CD103^−^ CD49a^+^ or CD69^+^ CD49a^+^ memory CD8^+^ T cells, did not have obvious fluctuation after reinfection. This observation was parallel with the finding in the pseudotime trajectory that CD49a expressing resident memory CD8^+^ T cells may have the potential to develop into the effector phenotype.

In the following section, we will describe the CCL signaling pathways discussed in the cell–cell interactions analysis during reinfection. As expected, reinfection induced chemokines response in the lungs. The mRNA expression of Ccl5 in lung tissue increased greatly after 3 days post-reinfection (Supplementary Figure 3C). Correspondingly, Ccl5 levels in the lung remained stable after reinfection, while Ccr5 and Ccl4 levels increased rapidly (Supplementary Figure 3D). We further investigated the expression of Ccl5 and Ccr5 in CD8^+^ Trm and CD69^+^ CD103^−^ cells, as well as in other subsets after reinfection. Our analysis revealed that Ccr5 expression remained high in CD8^+^ Trm cells after reinfection and was higher in CD8^+^ Trm cells than in other cells (Figure 6D). Moreover, Ccl5 expression in CD8^+^ Trm and CD69^+^ CD103^−^ cells increased at 1 day post-reinfection, while it remained low in CD8^+^ T cells from circulation (Figure 6D). This accumulating evidence might indicate that the Ccr5-Ccl5 pair played an important role in responses against reinfection and that it is more highly expressed in CD8^+^ Trm cells than other subsets with elevated Ccl5 signal after reinfection.

## 4. Discussion

Resident phenotypic memory CD8^+^ T cells play a key role in the defense against pathogens re-encountered in the lung. However, little is known about the potential transitions and regulation mechanisms of their function after influenza virus infection and re-exposure. In this study, we performed integrated transcriptome data analysis and *in vivo* experiments. We found that resident memory CD8^+^ T cells accounted for an increased ratio of all CD8^+^ T cells in the lung at the early recovery stage after influenza infection (Figure 1A). However, we observed a decline in both the absolute number and proportion of these cells over time (Figure 6B). This finding is in line with previous research results, suggesting that airway and lung parenchymal CD8^+^ Trm exhibit a proapoptotic phenotype and have an exceptionally short half-life (12 days in mice; Pizzolla et al., 2017; Zheng and Wakim, 2022). Moreover, we observed that CD8^+^ Trm cells expressed higher levels of *Itgae* (CD103) and *Cdh1* at D14 after infection compared to D7. CD103 and CDH1 are involved in regulating the cell–cell adhesions of epithelial cells. In addition, we noticed that the PD-1 expression pathway, which is crucial for the proliferation and maintenance of Trm cells (Li et al., 2019), was enriched in CD8^+^ Trm cells on day 14. One study also reveals that PD-1 expression by a population of lung Trm cells helps limit their inflammatory actions that might contribute to fibrosis (Wang et al., 2019).

We identified that CD8^+^ Trm cells with canonical markers CD69 and CD103 also expressed CD49a, which is consistent with previous research (Reilly et al., 2020). The expression of CD49a increased gradually at the early recovery stage after influenza infection (Supplementary Figure 1C) and remained at a high level over time (Figure 6B). CD49a facilitates the locomotion of virus-specific CD8^+^ T cells and may contribute to the local surveillance function of the Trm population (Reilly et al., 2020). Interestingly, a recent study revealed that CD103^−^-deficient CD8^+^ T cells in mouse skin after herpes simplex virus infection also displayed increased locomotion speed, suggesting that CD103 may restrain the cells' motility (Zaid et al., 2017). Furthermore, cells expressing both CD103 and CD49a showed the highest levels of effector responses, T-cell survival genes, spanning antiviral cytokines, chemokines, and cytolytic mediators compared with other memory T subsets after influenza infection (Reilly et al., 2021).

Our observation suggested a potential development from CD8^+^ Tem to Trm cells in the memory phase based on the pseudotime trajectory. It has been revealed that circulating memory CD8^+^ T cells can be recruited into the lung and converted into Trm phenotype without antigen recognition (Van Braeckel-Budimir and Harty, 2017). Airway T cells are mainly replenished by recruitment from the lung interstitium (Ely et al., 2006), and Trm cells in the interstitium receive continuous compensation from circulating Tem cells (Qian et al., 2020). However, several studies mentioned that the lung Trm compartment was not replaced by the circulating memory pool (Takamura et al., 2016; Van Braeckel-Budimir and Harty, 2017).

Meanwhile, we observed that resident memory CD8^+^ T subpopulations, including canonical Trm and subsets with high CD49a expression, were predominant during the memory phase and exhibited the potential to differentiate into effector types upon reinfection. The ratio of these resident phenotypic cells decreased after reinfection, which may parallel their potential transition into effector types. These results imply that lung resident memory T cells function as sentinels in the secondary immune response (Qian et al., 2020). During the first phase of the recall response, memory T cells residing in the tissue encounter the pathogen and initiate antiviral responses (Sallusto et al., 1999; Hikono et al., 2006). Additionally, we observed the upregulation of the type I interferon signaling pathway in CD8^+^ Trm cells after reinfection. Recent studies have demonstrated that the early response to type I interferons is necessary for the proper expansion and function of lung resident CD8^+^ memory T cells (Kohlmeier et al., 2010; Varese et al., 2022). Collectively, these findings suggest that the transition from a resident to an effector phenotype may be a critical aspect of CD8^+^ resident memory T cell defense against influenza infection, highlighting their importance as a first line of defense. However, further investigations are required to fully understand this phenomenon.

Furthermore, our results also showed that the PI3K-Akt-mTOR signaling pathway was enriched in CD8^+^ Tem cells after reinfection. Meanwhile, mTOR (mammalian target of rapamycin) reprograms metabolic changes in Trm cells (Jones and Pearce, 2017). Activated PI3K-Akt-mTOR pathway can improve T lymphocyte metabolism, nutrient uptake, and energy production, regulate cell cycle and apoptosis, and affect T lymphocyte activation and immune function (Liu et al., 2020). The differentiation into effector and memory CD8^+^ T cells is coordinated by the phosphoinositide 3-kinase (PI3K)/Akt pathway (Kim and Suresh, 2013). One study revealed that Akt activation regulated the differentiation of CD8^+^ T cells into effector and memory T cells, where sustained Akt activation led to the terminal differentiation of effector CD8^+^ T cells, whereas the inhibition of Akt *in vivo* increased the number of memory CD8^+^ T cells (Kim et al., 2012). Our findings suggest that CD8^+^ Trm cells have the developmental potential to convert to the effector phenotype after reinfection, and we speculate that the PI3K-Akt-mTOR pathway may promote the response of memory CD8^+^ T cells against reinfection.

The Ccl5-Ccr5 and Ccl4-Ccr5 ligand/receptor pairs were found to be important signaling pathways received by CD8^+^ Trm after reinfection in the present study. The study also revealed strengthened interactions of the Ccl5-Ccr5, Ccl4-Ccr5, and Ccl3-Ccr5 ligand/receptor pairs between CD8^+^ Trm and other memory subsets after influenza infection. One study suggested that the maintenance of CD4^+^ Trm cells within niches might require the chemokine Ccl5 from antigen-presenting or CD8^+^ T cells (Iijima and Iwasaki, 2014; Collins et al., 2016). Previous research showed that the rapid recruitment of memory CD8^+^ T cells to the lung airways were dependent on Ccr5 expression after secondary influenza challenge (Kohlmeier et al., 2008). A study of human lung samples showed that genes of CXCR3, CXCR6, and CCR5 have differential expression in lung Trm cells relative to that in blood-derived Tem cells (Hombrink et al., 2016). Our results also revealed that the expression of Ccr5 and Ccl5 in lung memory CD8^+^ T cells was higher than these cells in circulation (CD8b^+^) during reinfection. These data suggest the importance of Ccr5 interaction with related ligands in the maintenance and recruitment of resident memory CD8^+^ T cells after influenza infection and reinfection. However, the mechanisms are not fully understood.

Our study has several limitations that should be acknowledged. First, our findings are constrained by the restricted datasets and a limited number of CD8^+^ T cells analyzed. However, we believe that our study is still meaningful in providing a more comprehensive understanding of memory CD8^+^ T cells during influenza infection. In addition, it still needs more experimental data to further validate our findings in the present study. In addition, the utilization of classical gene markers derived from phenotypic research to elucidate CD8^+^ T cell subsets and functional states in the scRNA-seq data may have introduced certain biases. To address this, we selected markers carefully and referred to other studies that have employed scRNA-seq analysis (Szabo et al., 2019; Chen et al., 2021). We consider that some unidentified subsets might represent transitional stages of specific subsets and may help us to better understand the functions of CD8^+^ T cells. Finally, while immune infiltration analysis is mainly used in cancer studies, we used it to evaluate the proportion of immune cells in infectious diseases. Nevertheless, many studies have already employed this method in inflammatory diseases (Cao et al., 2019b; Kawada et al., 2021) and influenza samples (Lin et al., 2021; Sun et al., 2021).

## 5. Conclusion

Our data suggested that CD8^+^ Trm cells accumulated in the lung at the early recovery stage after influenza infection but declined quickly over time. Nearly half of these CD8^+^ Trm cells co-expressed CD49a. Resident memory CD8^+^ T cells could be reactivated rapidly against reinfection with the potential to differentiate into effector type upon reinfection. Functional differences existed in CD8^+^ Trm and Tem cells after influenza infection and reinfection. We also identified that Ccr5 with related ligand pairs was important in cell interaction between CD8^+^ Trm and other memory subsets after influenza infection. Additionally, the PI3K-Akt-mTOR and type I interferon signaling pathways were important for memory CD8^+^ T cells against reinfection. This study provides a comprehensive landscape for investigating the potential functional transition and regulating factors of resident memory CD8^+^ T after influenza infection and reinfection.

## Code availability

The R and custom scripts used for data analysis are available upon request. For further inquiries, please contact the corresponding author and the first author via email.

## Data availability statement

Publicly available datasets were analyzed in this study. This data can be found at: Gene Expression Omnibus (GEO) repository, https://www.ncbi.nlm.nih.gov/geo/ (GEO database: GSE183890, GSE186839, and GSE194058).

## Ethics statement

The animal study was reviewed and approved by Chinese Center for Disease Control and Prevention.

## Author contributions

BC: conception and design. JJ and HL: analysis and interpretation of data. JJ: performance of the experiments and drafting of the manuscript. YZ: revision of the manuscript. ZH and JY: interpretation of data and revision of the manuscript. All authors contributed to the manuscript and approved the submitted version.

## References

[B1] Ariotti S. Hogenbirk M. A. Dijkgraaf F. E. Visser L. L. Hoekstra M. E. Song J. Y.. (2014). T cell memory. Skin-resident memory CD8? T cells trigger a state of tissue-wide pathogen alert. Science 346, 101–5. 10.1126/science.1254803 25278612

[B2] Barbie D. A. Tamayo P. Boehm J. S. Kim S. Y. Moody S. E. Dunn I. F.. (2009). Systematic RNA interference reveals that oncogenic KRAS-driven cancers require TBK1. Nature 462, 108–112. 10.1038/nature08460 19847166 PMC2783335

[B3] Belz G. T. Xie W. Altman J. D. Doherty P. C. (2000). A previously unrecognized H-2D(b)-restricted peptide prominent in the primary influenza A virus-specific CD8(+) T-cell response is much less apparent following secondary challenge. J. Virol. 74, 3486–3493. 10.1128/JVI.74.8.3486-3493.2000 10729122 PMC111856

[B4] Cao J. Spielmann M. Qiu X. Huang X. Ibrahim D. M. Hill A. J.. (2019a). The single-cell transcriptional landscape of mammalian organogenesis. Nature 566, 496–502. 10.1038/s41586-019-0969-x 30787437 PMC6434952

[B5] Cao Y. Tang W. Tang W. (2019b). Immune cell infiltration characteristics and related core genes in lupus nephritis: results from bioinformatic analysis. BMC Immunol. 20, 37. 10.1186/s12865-019-0316-x 31638917 PMC6805654

[B6] Charoentong P. Finotello F. Angelova M. Mayer C. Efremova M. Rieder D.. (2017). Pan-cancer immunogenomic analyses reveal genotype-immunophenotype relationships and predictors of response to checkpoint blockade. Cell Rep. 18, 248–262. 10.1016/j.celrep.2016.12.019 28052254

[B7] Chen Y. Shen J. Kasmani M. Y. Topchyan P. Cui W. (2021). Single-cell transcriptomics reveals core regulatory programs that determine the heterogeneity of circulating and tissue-resident memory CD8(+) T cells. Cells 10, 82143. 10.3390/cells10082143 34440912 PMC8392357

[B8] Collins N. Jiang X. Zaid A. Macleod B. L. Li J. Park C. O.. (2016). Skin CD4(+) memory T cells exhibit combined cluster-mediated retention and equilibration with the circulation. Nat. Commun. 7, 11514. 10.1038/ncomms11514 27160938 PMC4866325

[B9] Ely K. H. Cookenham T. Roberts A. D. Woodland D. L. (2006). Memory T cell populations in the lung airways are maintained by continual recruitment. J. Immunol. 176, 537–543. 10.4049/jimmunol.176.1.537 16365448

[B10] Hasan M. H. Beura L. K. (2022). Cellular interactions in resident memory T cell establishment and function. Curr. Opin. Immunol. 74, 68–75. 10.1016/j.coi.2021.10.005 34794039 PMC8901561

[B11] Hikono H. Kohlmeier J. E. Ely K. H. Scott I. Roberts A. D. Blackman M. A.. (2006). T-cell memory and recall responses to respiratory virus infections. Immunol. Rev. 211, 119–132. 10.1111/j.0105-2896.2006.00385.x 16824122

[B12] Hombrink P. Helbig C. Backer R. A. Piet B. Oja A. E. Stark R.. (2016). Programs for the persistence, vigilance and control of human CD8(+) lung-resident memory T cells. Nat. Immunol. 17, 1467–1478. 10.1038/ni.3589 27776108

[B13] Iijima N. Iwasaki A. (2014). T cell memory. A local macrophage chemokine network sustains protective tissue-resident memory CD4 T cells. Science 346, 93–98. 10.1126/science.1257530 25170048 PMC4254703

[B14] Jin S. Guerrero-Juarez C. F. Zhang L. Chang I. Ramos R. Kuan C. H.. (2021). Inference and analysis of cell-cell communication using CellChat. Nat. Commun. 12, 1088. 10.1038/s41467-021-21246-9 33597522 PMC7889871

[B15] Jones R. G. Pearce E. J. (2017). MenTORing immunity: mTOR signaling in the development and function of tissue-resident immune cells. Immunity 46, 730–742. 10.1016/j.immuni.2017.04.028 28514674 PMC5695239

[B16] Kawada J. I. Takeuchi S. Imai H. Okumura T. Horiba K. Suzuki T.. (2021). Immune cell infiltration landscapes in pediatric acute myocarditis analyzed by CIBERSORT. J. Cardiol. 77, 174–178. 10.1016/j.jjcc.2020.08.004 32891480

[B17] Kim E. H. Sullivan J. A. Plisch E. H. Tejera M. M. Jatzek A. Choi K. Y.. (2012). Signal integration by Akt regulates CD8 T cell effector and memory differentiation. J. Immunol. 188, 4305–4314. 10.4049/jimmunol.1103568 22467649 PMC3331885

[B18] Kim E. H. Suresh M. (2013). Role of PI3K/Akt signaling in memory CD8 T cell differentiation. Front. Immunol. 4, 20. 10.3389/fimmu.2013.00020 23378844 PMC3561661

[B19] Kohlmeier J. E. Cookenham T. Roberts A. D. Miller S. C. Woodland D. L. (2010). Type I interferons regulate cytolytic activity of memory CD8(+) T cells in the lung airways during respiratory virus challenge. Immunity 33, 96–105. 10.1016/j.immuni.2010.06.016 20637658 PMC2908370

[B20] Kohlmeier J. E. Miller S. C. Smith J. Lu B. Gerard C. Cookenham T.. (2008). The chemokine receptor CCR5 plays a key role in the early memory CD8+ T cell response to respiratory virus infections. Immunity 29, 101–113. 10.1016/j.immuni.2008.05.011 18617426 PMC2519120

[B21] Kumar B. V. Ma W. Miron M. Granot T. Guyer R. S. Carpenter D. J.. (2017). Human tissue-resident memory T cells are defined by core transcriptional and functional signatures in lymphoid and mucosal sites. Cell Rep. 20, 2921–2934. 10.1016/j.celrep.2017.08.078 28930685 PMC5646692

[B22] Lange J. Rivera-Ballesteros O. Buggert M. (2022). Human mucosal tissue-resident memory T cells in health and disease. Mucosal Immunol. 15, 389–397. 10.1038/s41385-021-00467-7 34743182 PMC8571012

[B23] Li C. Zhu B. Son Y. M. Wang Z. Jiang L. Xiang M.. (2019). The transcription factor Bhlhe40 programs mitochondrial regulation of resident CD8(+) T cell fitness and functionality. Immunity 51, 491–507.e7. 10.1016/j.immuni.2019.08.013 31533057 PMC6903704

[B24] Lin S. Peng Y. Xu Y. Zhang W. Wu J. Zhang W.. (2021). Establishment of a risk score model for early prediction of severe H1N1 influenza. Front. Cell Infect. Microbiol. 11, 776840. 10.3389/fcimb.2021.776840 35059324 PMC8764189

[B25] Liu Y. Liu S. Wu C. Huang W. Xu B. Lian S.. (2020). PD-1-mediated PI3K/Akt/mTOR, caspase 9/caspase 3 and ERK pathways are involved in regulating the apoptosis and proliferation of CD4(+) and CD8(+) T cells during BVDV infection *in vitro*. Front. Immunol. 11, 467. 10.3389/fimmu.2020.00467 32256500 PMC7089960

[B26] MacLean A. J. Richmond N. Koneva L. Attar M. Medina C. A. P. Thornton E. E.. (2022). Secondary influenza challenge triggers resident memory B cell migration and rapid relocation to boost antibody secretion at infected sites. Immunity 55, 718–733.e8. 10.1016/j.immuni.2022.03.003 35349789 PMC9044924

[B27] Oja A. E. Piet B. Helbig C. Stark R. van der Zwan D. Blaauwgeers H.. (2018). Trigger-happy resident memory CD4(+) T cells inhabit the human lungs. Mucosal Immunol. 11, 654–667. 10.1038/mi.2017.94 29139478

[B28] Paules C. I. Sullivan S. G. Subbarao K. Fauci A. S. (2018). Chasing seasonal influenza—The need for a universal influenza vaccine. N. Engl. J. Med. 378, 7–9. 10.1056/NEJMp1714916 29185857

[B29] Pizzolla A. Nguyen T. H. O. Smith J. M. Brooks A. G. Kedzieska K. Heath W. R.. (2017). Resident memory CD8(+) T cells in the upper respiratory tract prevent pulmonary influenza virus infection. Sci. Immunol. 2, aam6970. 10.1126/sciimmunol.aam6970 28783656

[B30] Pizzolla A. Wakim L. M. (2019). Memory T cell dynamics in the lung during influenza virus infection. J. Immunol. 202, 374–381. 10.4049/jimmunol.1800979 30617119

[B31] Qian Y. Zhu Y. Li Y. Li B. (2020). Legend of the sentinels: development of lung resident memory T cells and their roles in diseases. Front. Immunol. 11, 624411. 10.3389/fimmu.2020.624411 33603755 PMC7884312

[B32] Reilly E. C. Lambert Emo K. Buckley P. M. Reilly N. S. Smith I. Chaves F. A.. (2020). T(RM) integrins CD103 and CD49a differentially support adherence and motility after resolution of influenza virus infection. Proc. Natl. Acad. Sci. U. S. A. 117, 12306–12314. 10.1073/pnas.1915681117 32439709 PMC7275699

[B33] Reilly E. C. Sportiello M. Emo K. L. Amitrano A. M. Jha R. Kumar A. B. R.. (2021). CD49a identifies polyfunctional memory CD8 T cell subsets that persist in the lungs after influenza infection. Front. Immunol. 12, 728669. 10.3389/fimmu.2021.728669 34566986 PMC8462271

[B34] Sallusto F. Lenig D. Förster R. Lipp M. Lanzavecchia A. (1999). Two subsets of memory T lymphocytes with distinct homing potentials and effector functions. Nature 401, 708–712. 10.1038/44385 10537110

[B35] Schenkel J. M. Fraser K. A. Vezys V. Masopust D. (2013). Sensing and alarm function of resident memory CD8? T cells. Nat. Immunol. 14, 509–513. 10.1038/ni.2568 23542740 PMC3631432

[B36] Schmidt M. E. Varga S. M. (2018). The CD8 T cell response to respiratory virus infections. Front. Immunol. 9, 678. 10.3389/fimmu.2018.00678 29686673 PMC5900024

[B37] Sun B. Guo X. Wen X. Xie Y. B. Liu W. H. Pang G. F.. (2021). Application of weighted gene co-expression network analysis to identify the hub genes in H1N1. Int. J. Physiol. Pathophysiol. Pharmacol. 13, 69–85. 34336131 PMC8310883

[B38] Szabo P. A. Levitin H. M. Miron M. Snyder M. E. Senda T. Yuan J.. (2019). Single-cell transcriptomics of human T cells reveals tissue and activation signatures in health and disease. Nat. Commun. 10, 4706. 10.1038/s41467-019-12464-3 31624246 PMC6797728

[B39] Takamura S. Yagi H. Hakata Y. Motozono C. McMaster S. R. Masumoto T.. (2016). Specific niches for lung-resident memory CD8+ T cells at the site of tissue regeneration enable CD69-independent maintenance. J. Exp. Med. 213, 3057–3073. 10.1084/jem.20160938 27815325 PMC5154946

[B40] Topham D. J. Reilly E. C. (2018). Tissue-resident memory CD8(+) T cells: from phenotype to function. Front. Immunol. 9, 515. 10.3389/fimmu.2018.00515 29632527 PMC5879098

[B41] Van Braeckel-Budimir N. Harty J. T. (2017). Influenza-induced lung T(rm): not all memories last forever. Immunol. Cell Biol. 95, 651–655. 10.1038/icb.2017.32 28405016

[B42] van de Wall S. Badovinac V. P. Harty J. T. (2021). Influenza-specific lung-resident memory CD8(+) T cells. Cold Spring Harb. Perspect. Biol. 13, a037978. 10.1101/cshperspect.a037978 33288540 PMC7849341

[B43] Varese A. Nakawesi J. Farias A. Kirsebom F. C. M. Paulsen M. Nuriev R.. (2022). Type I interferons and MAVS signaling are necessary for tissue resident memory CD8+ T cell responses to RSV infection. PLoS Pathog. 18, e1010272. 10.1371/journal.ppat.1010272 35108347 PMC8843175

[B44] Wang Z. Wang S. Goplen N. P. Li C. Cheon I. S. Dai Q.. (2019). PD-1(hi) CD8(+) resident memory T cells balance immunity and fibrotic sequelae. Sci. Immunol. 4, aaw1217. 10.1126/sciimmunol.aaw1217 31201259 PMC7458867

[B45] Wu T. Hu Y. Lee Y. T. Bouchard K. R. Benechet A. Khanna K.. (2014). Lung-resident memory CD8 T cells (TRM) are indispensable for optimal cross-protection against pulmonary virus infection. J. Leukoc. Biol. 95, 215–224. 10.1189/jlb.0313180 24006506 PMC3896663

[B46] Yewdell J. W. Bennink J. R. Smith G. L. Moss B. (1985). Influenza A virus nucleoprotein is a major target antigen for cross-reactive anti-influenza A virus cytotoxic T lymphocytes. Proc. Natl. Acad. Sci. U. S. A. 82, 1785–1789. 10.1073/pnas.82.6.1785 3872457 PMC397357

[B47] Yu G. Wang L. G. Han Y. He Q. Y. (2012). clusterProfiler: an R package for comparing biological themes among gene clusters. Omics 16, 284–287. 10.1089/omi.2011.0118 22455463 PMC3339379

[B48] Yuan R. Yu J. Jiao Z. Li J. Wu F. Yan R.. (2021). The roles of tissue-resident memory T cells in lung diseases. Front. Immunol. 12, 710375. 10.3389/fimmu.2021.710375 34707601 PMC8542931

[B49] Zaid A. Hor J. L. Christo S. N. Groom J. R. Heath W. R. Mackay L. K.. (2017). Chemokine receptor-dependent control of skin tissue-resident memory T cell formation. J. Immunol. 199, 2451–2459. 10.4049/jimmunol.1700571 28855310

[B50] Zheng M. Z. M. Wakim L. M. (2022). Tissue resident memory T cells in the respiratory tract. Mucosal Immunol. 15, 379–388. 10.1038/s41385-021-00461-z 34671115 PMC8526531

[B51] Zou J. Deng F. Wang M. Zhang Z. Liu Z. Zhang X.. (2022). scCODE: an R package for data-specific differentially expressed gene detection on single-cell RNA-sequencing data. Brief Bioinform. 23, bbac180. 10.1093/bib/bbac180 35598331

