# Supplementary material for: Comprehensive immune landscape of lung-resident memory CD8+ T cells after influenza infection and reinfection in a mouse model
Source: Front Microbiol. 2023 Jun 21;14:1184884. doi: 10.3389/fmicb.2023.1184884 (PMC10320391; doi:10.3389/fmicb.2023.1184884)
Supplement: Supplementary file 1 [file Data_Sheet_1.doc]

**Supplementary information**

**Title:**

Comprehensive immune landscape of lung-resident memory CD8^+^ T cells after influenza infection and reinfection in a mouse model

**Catalogue:**

1. Supplementary methods

2. Supplementary Figures

3. Supplementary Tables

4. List of abbreviations

**Supplementary methods**

1. **Data collection and preprocessing**

Because dataset GSE183890 contained 22 IFITM3-KO and 24 normal lung samples, we selected 12 normal samples for investigation. They were divided and processed in 2 groups including 3 days post primary infection with PR8 H1N1 virus (mock_PR8challenge_3dpi) and 3 days post reinfection with PR8 (PR8_PR8challenge_3dpi). In addition, the mice in this dataset were reinfected four weeks after primary infection and other detailed information was shown in Supplementary Table 2.

1. **scRNA-seq data processing**

Cells expressing fewer than 200 genes or genes expressed in fewer than 3 cells were discarded. Next, we filtered cells that had less than 200 or more than 5000 features, and those with a mitochondrial RNA percentage greater than 10 or ribosome RNA percentage less than 3 were also removed. The Harmony algorithm [1] was used to integrate the data, and DoubletFinder [2] to perform doublet analysis sequentially.

1. **Analysis of infiltrating immune cells**

The ssGSEA algorithm [3] is a modification of the standard Gene Set Enrichment Analysis (GSEA) method. And the difference is that ssGSEA considers each sample individually, without the need for a comparison group and calculates an enrichment score for each sample and gene set, which reflects the degree to which the genes in the set are coordinately upregulated or downregulated in that sample. This algorithm was published about fifteen years ago [3], And has performed to estimate the relative composition of different immune infiltrating cells based on mRNA expression data in immune gene sets [4; 5].

1. **RNA extraction and RT-PCR analysis**

Lungs obtained after influenza infection and frozen in liquid nitrogen were homogenized using a Lysing Matrix M tube with a FastPrep-24TM instrument. RNA was extracted from the homogenized lung tissue using the MiniBEST Universal RNA Extraction Kit (Takara). One microgram of purified RNA was then reversely transcribed into cDNA using the PrimeScript™ RT Master Mix (Takara). RT-PCR analysis was performed using SYBR Green on an Applied Biosystems QuantStudio® 12K Flex Real Time PCR thermocycler (Life Technologies™).

**Supplementary Figures**


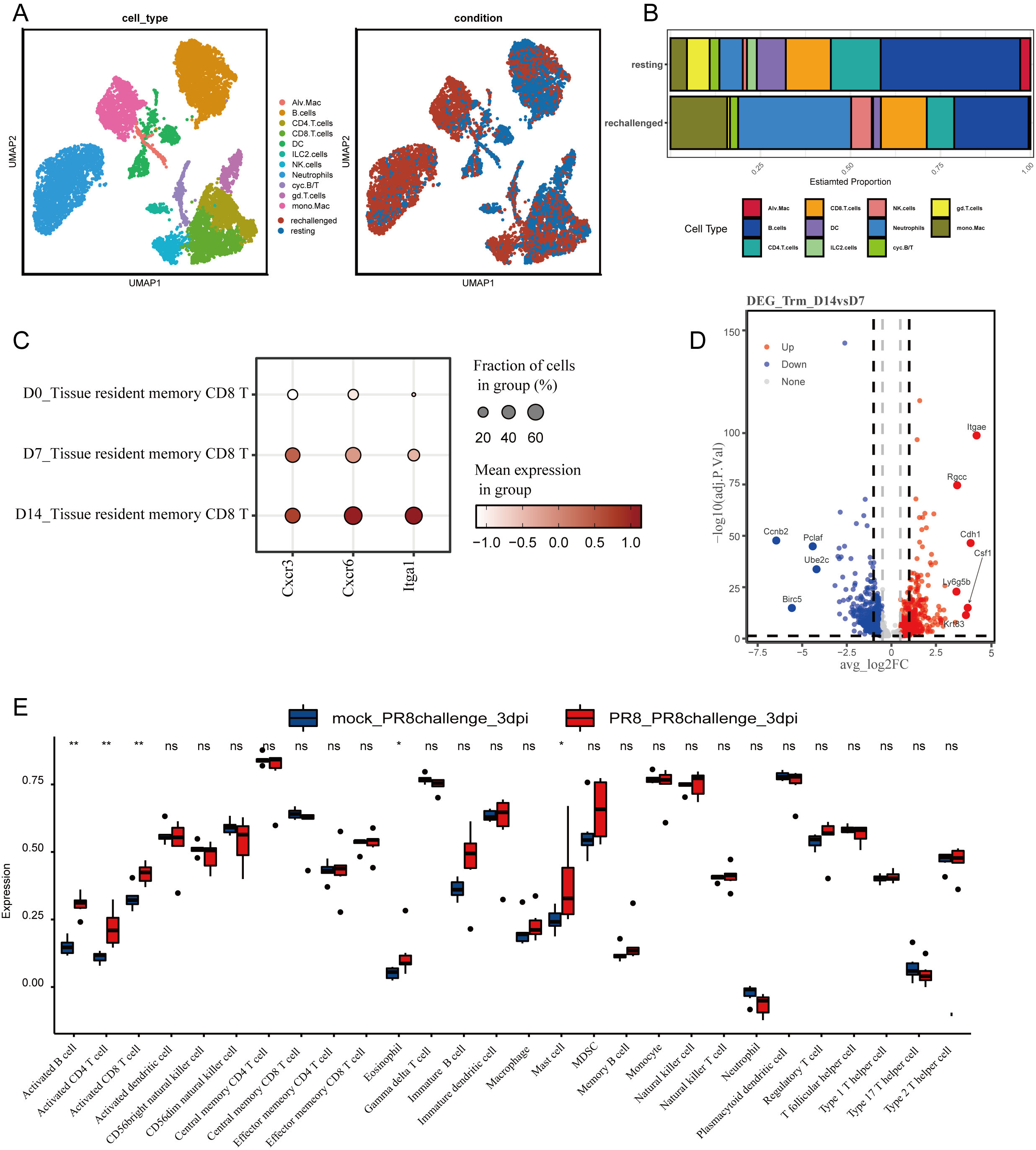


Supplementary Figure1. Supplementary plots for Figure1, Figure2 and Figure3. (A) UMAP plots presented main cell types (Left panel) and the composition of cells in resting and rechallenged (right panel) processed in published research about GSE194058. (B) The bar plot displayed the proportion of cell types mentioned in A, separated by conditions. (C) The plot presented the representative differentially expressed genes, including *Cxcr3,* *Cxcr6* and *Itga1* in CD8^+^ Trm cells during different conditions in GSE186839. (D) The volcano plot displayed the DEGs of Trm in D14 compared to D7. Red dots represent genes upregulated in D14. (E) The box plot exhibited the difference in immune cell fractions between PR8_PR8challenge_3dpi and mock_PR8challenge_3dpi samples.


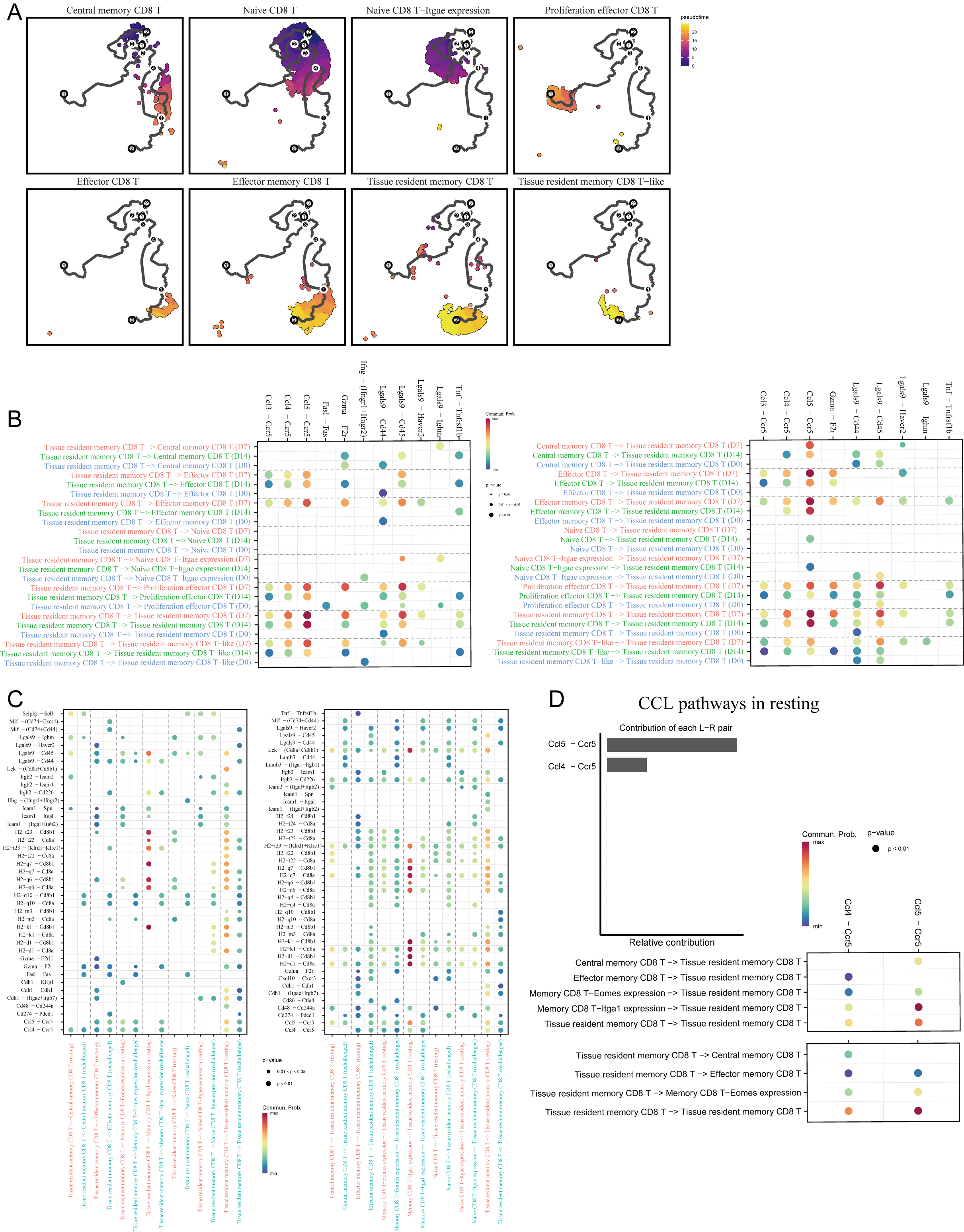


Supplementary Figure2. Trajectory and CellChat analysis performed among memory CD8^+^ T cells. (A) The UMAPs showed pseudotime trajectory of CD8^+^ T cell subsets in D0, D7 and D14, with cells colored according to predicted pseudotime. (B) The dotplot showed the significant ligand-receptor pairs that contributed to the signaling from Trm cells to other subpopulations (left panel) and from other CD8^+^ T cell subsets to Trm cells (right panel) in D0, D7 and D14 conditions. Dot color reflected communication probabilities and dot size represented computed p-values, with empty space indicating a zero communication probability. The p-values were computed from one-sided permutation test. (C) The dotplot showed the significant ligand-receptor pairs in resting and rechallenged conditions. (D)The plots showed the relative contribution of CCL pathways (top) and communication probabilities mediated by CCL ligand-receptor pairs between CD8^+^ Trm and other subsets (bottom) in rechallenged.


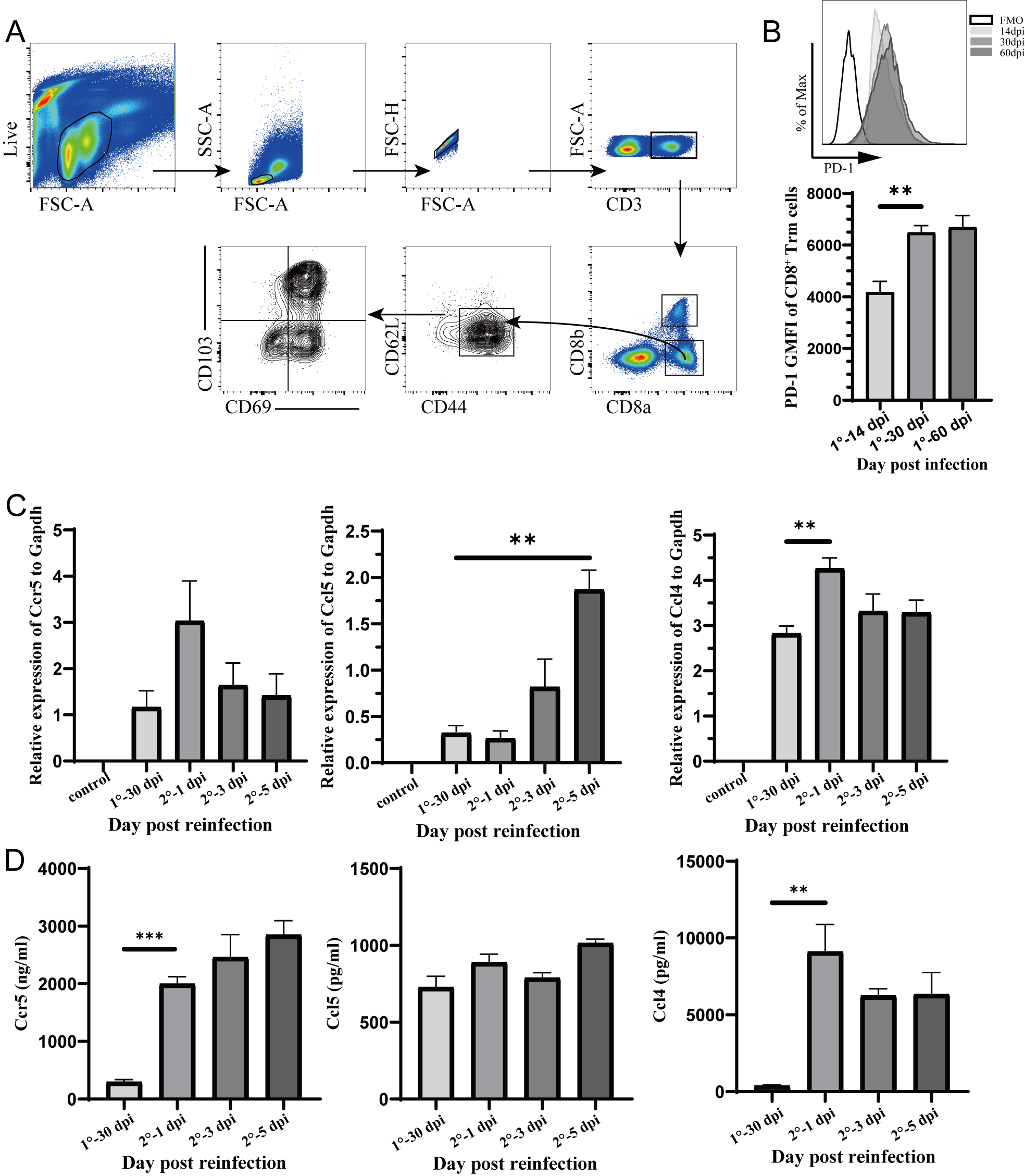


Supplementary Figure3. Validation of observations in this study relevant to influenza infection and reinfection. (A) Gating strategy of representative flow cytometry plots displayed surface staining of CD3, CD8a, CD8b, CD44, CD62L, CD69 and CD103 in lung cells from mice. (B) The expression of PD-1 was evaluated in CD8^+^ Trm cells from the lung (iv. CD8b^-^) at 14, 30, and 60 days post infection. GMFI represented Geometric Mean Fluorescence Intensity. The data were presented as mean±SEM of 5 PR8 mice in every group. (C) mRNA expression of Ccr5, Ccl5 and Ccl4 in lung samples were quantified by RT-PCR . (D) Ccr5, Ccl5 and Ccl4 levels in lung samples were analyzed by ELISA. Data were presented as mean±SEM of 3 mice in each group. 1° represented primary infection; 2° represented reinfection; dpi meant day post infection. Statistical significances were analyzed through unpaired two-tailed Student’s t-test. **P* < 0.05, ***P* < 0.01, ****P* < 0.001, and *****P* < 0.0001.

**Supplementary Tables**

Supplementary Table 1. Characteristics of involved samples in GSE datasets.

| **GEO number** | **Type** | **Sample quantity** | **Species** | **Tissue** | **Treatment** | **Time point for samples collecting** |
| --- | --- | --- | --- | --- | --- | --- |
| GSE186839 | scRNA-seq | 3 | Mus musculus | Lung | IAV | 0, 7, 14 days post infection |
| GSE194058 | scRNA-seq | 3 | Mus musculus | Lung | X31 influenza virus | 42 days post infection and one day after rechallenge |
| GSE183890 | Bulk RNA-Seq | 46 | Mus musculus | Lung | PR8 H1N1 and X31 H3N2 influenza virus | 3 days post primary and secondary infection with PR8 H1N1; 3 or 5 days post reinfection with X31 H3N2 |

Supplementary Table 2. Characteristics of samples used in this study in GSE183890

| **Sample ID** | **Tissue** | **Sex** | **Primary infection strain** | **Reinfection strain** | **Days post infection** | **Clinical traits** |
| --- | --- | --- | --- | --- | --- | --- |
| IFITM3_7 | Lung | Female | PR8 | PR8 | 3 | PR8_PR8challenge_3dpi |
| IFITM3_8 | Lung | Female | PR8 | PR8 | 3 | PR8_PR8challenge_3dpi |
| IFITM3_9 | Lung | Female | PR8 | PR8 | 3 | PR8_PR8challenge_3dpi |
| IFITM3_10 | Lung | Male | PR8 | PR8 | 3 | PR8_PR8challenge_3dpi |
| IFITM3_11 | Lung | Male | PR8 | PR8 | 3 | PR8_PR8challenge_3dpi |
| IFITM3_12 | Lung | Male | PR8 | PR8 | 3 | PR8_PR8challenge_3dpi |
| IFITM3_13 | Lung | Female | PR8 | / | 3 | mock_PR8challenge_3dpi |
| IFITM3_14 | Lung | Female | PR8 | / | 3 | mock_PR8challenge_3dpi |
| IFITM3_15 | Lung | Female | PR8 | / | 3 | mock_PR8challenge_3dpi |
| IFITM3_16 | Lung | Male | PR8 | / | 3 | mock_PR8challenge_3dpi |
| IFITM3_17 | Lung | Male | PR8 | / | 3 | mock_PR8challenge_3dpi |
| IFITM3_18 | Lung | Male | PR8 | / | 3 | mock_PR8challenge_3dpi |

Supplementary Table 3. The primer used in the RT-PCR

| **Primer** | **Sequence (5'-3')** |
| --- | --- |
| CCL5 | F：GTATTTCTACACCAGCAGCAAG |
|  | R：TCTTGAACCCACTTCTTCTCTG |
| CCL4 | F：TTCCTGCTGTTTCTCTTACACCT |
|  | R：CTGTCTGCCTCTTTTGGTCAG |
| CCR5 | F：ACTGCTGCCTAAACCCTGTC |
|  | R：ATGTTCTCCTGTGGATCGGG |
| GAPDH | Mouse GAPDH Endogenous Reference Genes Primers，100 μM obtained from Sangon Biotech (Shanghai) |

**List of abbreviations**

scRNA-seq single-cell RNA sequencing

Tcm central memory T

Tem effector memory T

Trm tissue resident memory T

bulk RNA-seq bulk RNA sequencing

GEO Gene Expression Omnibus

TPM transcripts per kilobase million

FACS fluorescence-activated cell sorting

ssGSEA single sample gene set enrichment analysis

WGCNA Weighted gene co-expression network analysis

PCA Principal Component Analysis

t-SNE T-Distribution Stochastic Neighbour Embedding

UMAP Uniform Manifold Approximation and Projection

DEGs Differentially expressed genes

GO Gene Ontology

KEGG Kyoto Encyclopedia of Genes and Genomes

GSEA Gene set enrichment analysis

GSVA Gene set variation analysis

Eff effector T cell

NT naive T cells

TF transcription factor

**References**

[1] I. Korsunsky, N. Millard, J. Fan, K. Slowikowski, F. Zhang, K. Wei, Y. Baglaenko, M. Brenner, P.R. Loh, and S. Raychaudhuri, Fast, sensitive and accurate integration of single-cell data with Harmony. Nat Methods 16 (2019) 1289-1296.

[2] C.S. McGinnis, L.M. Murrow, and Z.J. Gartner, DoubletFinder: Doublet Detection in Single-Cell RNA Sequencing Data Using Artificial Nearest Neighbors. Cell Syst 8 (2019) 329-337.e4.

[3] D.A. Barbie, P. Tamayo, J.S. Boehm, S.Y. Kim, S.E. Moody, I.F. Dunn, A.C. Schinzel, P. Sandy, E. Meylan, C. Scholl, S. Fröhling, E.M. Chan, M.L. Sos, K. Michel, C. Mermel, S.J. Silver, B.A. Weir, J.H. Reiling, Q. Sheng, P.B. Gupta, R.C. Wadlow, H. Le, S. Hoersch, B.S. Wittner, S. Ramaswamy, D.M. Livingston, D.M. Sabatini, M. Meyerson, R.K. Thomas, E.S. Lander, J.P. Mesirov, D.E. Root, D.G. Gilliland, T. Jacks, and W.C. Hahn, Systematic RNA interference reveals that oncogenic KRAS-driven cancers require TBK1. Nature 462 (2009) 108-12.

[4] J. Wang, Y. Liu, Y. Zhang, S. Wang, S. Kang, N. Mi, R. Li, and Y. Zou, Identification immune response genes in psoriasis after treatment with secukinumab. BMC Med Genomics 16 (2023) 77.

[5] B. Xiao, L. Liu, A. Li, C. Xiang, P. Wang, H. Li, and T. Xiao, Identification and Verification of Immune-Related Gene Prognostic Signature Based on ssGSEA for Osteosarcoma. Front Oncol 10 (2020) 607622.
